# Can an intervention integrating sports and medicine improve children’s health more effectively? Monitoring based on sleep, body mass index, and heart rate variability

**DOI:** 10.7189/jogh.14.04040

**Published:** 2024-04-19

**Authors:** Kun Wang, Yan Li, Shiqi Liu, Hengxu Liu, Tingran Zhang, Jiong Luo

**Affiliations:** 1 College of Physical Education, Southwest University, Research Centre for Exercise Detoxification, Chongqing, China; 2 College of Liberal Studies, Chongqing Industry Polytechnic College (Sports Work Department), Yubei, Chongqing, China

## Abstract

**Background:**

Theoretical studies have suggested that the integration of sports and medicine with one another could positively affect children’s health. By monitoring the variation characteristics of children’s sleep, body mass index (BMI), and heart rate variability (HRV), we explored and compared the influences of and differences between two interventions – physical exercise and an intervention integrating sports and medicine – on improving children's health.

**Methods:**

We conducted a randomised controlled study, where we randomly divided 136 children into the physical exercise group (PEG), the integration of sports and medicine group (ISMG), and the control group. We measured sleep, BMI, and HRV at baseline and week eight.

**Results:**

After the eight-week intervention, the sleep scores in the PEG and the ISMG were significantly lower than in the control group, while the scores in the ISMG were significantly lower than in the PEG. After the eight-week intervention, the BMI of both the PEG and the ISMG was significantly lower than that of the control group, without a significand difference between the two intervention groups. After the eight-week intervention, the standard deviation of normal-to-normal intervals (SDNN), root mean square differences of the standard deviation (RMSSD), low-frequency of normal (LFn), and high-frequency of normal (HFn) in the PEG and the ISMG were significantly higher than those in the control group, again without a significant difference between the two intervention groups. After intervention, sleep, BMI, and HRV of the three groups were correlated with one another to different degrees, but the correlation coefficient of the two exercise groups was higher.

**Conclusions:**

Based on the interventions, we observed a significant correlation between sleep, BMI, and HRV in children. Regular physical exercise or an intervention integrating sports and medicine could synergistically improve sleep, BMI, and HRV in this population, with the latter having a better effect on improving sleep quality.

In recent years, the lifestyle of Chinese children has been characterised by decreased sleep quality, decreased physical activity, and increased sedentary time, which has negatively affected their physical and mental health [1]. While sleep deficiency is common among children worldwide, it may pose a more significant problem in China [2]. Studies have shown that good sleep quality positively impacts children's physical and mental health [3], with adequate sleep being positively correlated with body growth and the development and the regulation of immune function [4], as well as positive emotions, academic performance, and quality of life [5]. Second, with improvements in diet, the increase in static life, and low physical activity, the childhood obesity rate has been increasing both in China [6,7] and globally [8]. Obesity not only harms the cardiovascular system and endocrine systems, but also affects the psychological behaviour and cognitive ability of children and adolescents [9,10] and profoundly impacts the quality of adult life [11]. More seriously, the occurrence of cardiovascular metabolic risk factors such as dyslipidaemia and hypertension in Chinese children and adolescents has been rising [12,13].

In relation to this, children’s sleep duration and sleep quality are closely related to obesity [14,15] and impaired cardiac autonomic nerve regulation [16]. Children with simple obesity are prone to reduced vagus nerve activity and an imbalance between the sympathetic and vagus nerve [17]. Considering this, we can see that children’s health is developing into a global public health problem, making interventions targeting their health level an important area of study.

Regular physical exercise plays a unique role in preventing chronic diseases and maintaining health. A recent study found that it can effectively improve sleep quality [18], with a meta-analysis indicating an overall moderate effect size [19]. This benefit was demonstrated in both patients with mental illness [20] and adolescents [21]. Simultaneously, insufficient physical exercise has been found to reduce energy consumption, which is likely to lead to obesity in children [22]. Studies have shown that physical exercise could prevent the early occurrence of obesity [23], help reduce children’s subcutaneous and visceral fat [24], and be an effective way to regulate body mass index (BMI). It can also enhance heart function, immune capacity, and improve insulin resistance [24], as well as regulate heart rate variability (HRV) [25]. Moreover, appropriate aerobic exercise could increase vagus nerve tone and induce adaptive changes in HRV [26], as confirmed by Villafaina et al. [27] in obese children and adolescents. These findings suggest that physical exercise has strong health promotion benefits and potential. However, for children who lack subjective initiative and exercise cognition, a single mode of physical exercise may make achieving the maximum benefit of chronic disease prevention difficult, whereby better results could be reached by combining other health promotion methods based on exercise intervention.

In October 2016, the Healthy China 2030 plan issued by the State Council of China highlighted a need to ‘strengthen the integration of sports and medicine and non-medical health intervention, and promote the formation of a disease management and health service model of integration of sports and medicine’ [28]. The ‘integration of sports and medicine’ advocates a new concept of transforming ‘medical health interventions’ into ‘sports health interventions’ and promotes a new ‘autonomous’ health intervention mode focussed on prevention [29]. Li et al. [30] proposed that it should be seen as the scientific integration of physical exercise and medicine, meaning the organic combination of different forms of physical exercise with modern medical concepts and medical technology methods. While exercise is seen as a method of improving one's body, medicine is seen as a method for maintaining one's health; consequently, the integration of these two approaches has had a proven effect on health promotion, disease prevention, treatment, and rehabilitation [31]. Therefore, the goal integrating sports and medicine together was to maximise their effect on health promotion through scientific interventions, especially to reduce or delay the occurrence of diseases [32].

However, these efforts began late in China and remain at the level of systematic theoretical construction, thereby still facing difficulties such as an imperfect core content system, unclear breakthrough of promotion and application, and unclear boundary between sports and medicine [30]. At the intervention level, the organic integration of sports and medicine in practice still needs in-depth exploration [33]. Meanwhile, the only empirical studies on the integration of sports and medicine focussed on college students [34] and female groups with abnormal blood pressure [35], with few reports on children. This holds special relevance as the physical health promotion and disease prevention of children and adolescents is one of the key tasks in deepening the integration of physical and medical services within the Healthy China plan [36]. Moreover, while physical medicine integration has been supported by many theoretical studies as a new method to improve public health, it lacks direct evidence from experimental studies. Based on this, by developing two intervention modes of ‘physical exercise’ and ‘integration of sports and medicine’, we aimed to investigate whether a physical and medical integration intervention is more beneficial to promoting children’s physical activity and mental health than physical exercise alone, but also to determine if there is a synergetic correlation between the measures after intervention. Our findings could provide reference for the development of evidence-based, effective intervention programmes for improving children’s health.

## METHODS

### Participants

We recruited 136 children from three classes at the Yongxiang Experimental Primary School in Chongqing, China, through the class number random method (where number 1 indicates class 1, the number 2 indicates class 2, etc.) and screened them according to pre-defined criteria. Specifically, we included children aged 9–12 years who agreed to participate, had no physical exercise habit, were of good overall health, and were able to participate in moderate or above intensity exercise. We excluded children with a history of heart disease or a history of mental illness.

We then randomly divided the participants into groups with the random number method (1 represents the physical exercise group (PEG), 2 represents the control group, etc.), with 45 people assigned to the PEG, 45 to the control group, and 46 into the integration of sports and medicine group (ISMG). Their demographic characteristics are shown in **Table 1**.

**Table 1:** The demographic data of the participants

Variable	PEG (n = 45), x̄ (SD)	ISMG (n = 46), x̄ (SD)	CG (n = 45), x̄ (SD)	F/χ^2^	*P*-value
Age in years					
*Rural*	10.95 (0.43)	11.13 (0.70)	10.98 (0.62)	1.10	>0.05
Body height in m					
*Rural*	1.48 (0.07)	1.49 (0.08)	1.51 (0.07)	2.08	>0.05
Body weight in kg					
*Rural*	38.73 (8.31)	39.10 (7.93)	39.12 (7.97)	0.03	>0.05
Place of residence, n (%)					
*Rural*	24 (53.33)	22 (47.83)	20 (44.44)	0.58	>0.05
*Urban*	21 (46.67)	24 (52.17)	25 (55.56)		
Monthly household income in CNY, n (%)					
*≤5000*	14 (31.11)	15 (32.61)	12 (26.67)	0.77	>0.05
*5000–10 000*	20 (44.44)	19 (41.30)	19 (42.22)		
*≥10000*	11 (24.44)	12 (26.09)	14 (31.11)		

CG – control group, ISMG – integration of sports and medicine group, PEG – physical exercise group, SD – standard deviation, x̄ – mean

The Ethics Committee of the School of Southwest University, China approved this study (SWU-TY202105). We followed the Declaration of Helsinki in conducting all procedures and obtained written informed consent from all participants, as well as approval of their parents who signed the parental informed consent.

### Intervention processes

The intervention for the PEG mainly included a preparation part (five minutes of warm-up activity), a basic part (30-minute exercise intervention (**Table 2**)), and an end part (five minutes stretching and relaxation activity). The exercise intervention programme was based on previous studies [34,38] and compiled by professional fitness coaches according to the children’s growth and development characteristics.

**Table 2:** List of intervention programmes*

Programmes	Intervention content	Load
Exercise programme	Aerobics calisthenics (classic step, step high five, classic parallel step, parallel up lift, stretch movement, balance movement, suction leg jump, cross squat, and left and right squat. All 4 × 8 beats).	The frequency was three sessions/week, the time was 40 min/session, and the intensity was moderate (65% to 75% HRmax).
	Resistance exercise (two people shoulder squat ×10, lunge squat 12 times, prone mountain climbing run 30s, and static straight arm push-ups 30s).	
	Jump rope 120 times alone.	
	50m serpentine run (50m turnback × 2).	
Medical programme	Health education lecture (such as the concept of integration of sports and medicine, common diseases and prevention methods of children, the harm of overweight and lack of sleep, the benefits of physical exercise, and health cognitive guidance).	The frequency of the ‘health education lecture’ was once a week and the time was 30min/session. Medical monitoring and supervision throughout the intervention.
	Medical monitoring and evaluation (exercise risk screening and evaluation, heart rate and blood pressure monitoring during exercise, and diagnosis and evaluation of post-exercise function).	
	Medical supervision (close monitoring of the medical staff during the exercise).	

*The setting of the HR_max_ standard was based on the previous study, that is, HR_max_ = 206.9 − 0.67 × age [37]. We monitored the heart rate during exercise using a Polar RCX3 heart rate meter.

**Figure 1:**
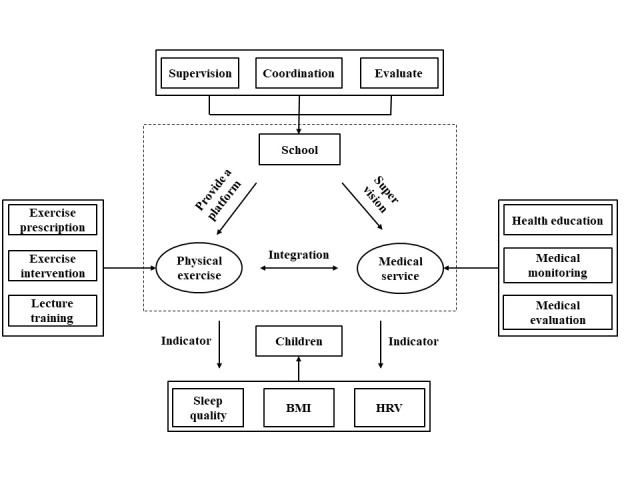
Flowchart of intervention integrating sports and medicine.

The content, time, and form of the physical exercise intervention for the ISMG were consistent with those of PEG. On this basis, we combined the physical exercise intervention with a medical intervention, including regular health education lectures, medical monitoring and evaluation, and medical supervision (**Figure 1** and **Table 2**). The design of the medical intervention programme fully followed the basic framework and logic of previous studies [39,40]. Specifically, this framework jointly integrates stakeholders in industry, academia, and research, as well as cutting-edge scientific knowledge and technology.

The control group did not participate in physical exercise or in the intervention integrating sports and medicine, but only rested or did extracurricular reading. For example, each time participants in the experimental group performed a corresponding intervention, participants in the control group were uniformly assigned to rest or read in the classroom, thereby avoiding additional physical activity or the medical intervention. To avoid affecting their academic performance, all participants had to normally complete the teaching courses and academic tasks required by the school.

### Measurement tools

We assessed sleep quality in the last month by the Pittsburgh Sleep Quality Index (PSQI) [41], which consists of 19 self-rated items and five externally rated items. However, the 19th self-rated item and the five other rated items are not calculated into the final score. The 18 self-assessment items are divided into seven dimensions: Sleep quality, sleep latency, sleep duration, sleep efficiency, sleep disorders, sleep medication use, and daytime dysfunction. Each component is scored on a scale of 0 to 3, and the score of each dimension is added together to obtain the total score of PSQI (0 to 21 points), with higher scores indicating worse sleep.

We measured the participants’ height and calculated their BMI per the formula BMI = weight in kilograms/(height in meters × height in meters).

We monitored and recorded changes in cardiac autonomic nerves by an HRV monitor (HeaLink-R211B Micro-ECG, China). We used the V5 guide to measure the Ag/AgCl single-use electrocardiogram (ECG) electrodes, with the bandwidth of the device at 0.5–40 Hz and the sampling frequency at 400 Hz. The acquisition indexes were composed of time-domain indexes and frequency-domain indexes, among which the former includes the standard deviation of normal-to-normal intervals (SDNN) and the root mean square differences of the standard deviation (RMSSD). The latter include low-frequency of normal (LFn) (LFn = (LF/(total power (TP) − very low frequency (VLF)) × 100%), high-frequency of normal (HFn) (HFn = (HF/(TP − VLF) × 100%)), and the ratio of low-frequency and high-frequency (LF/HF). During the test, the participants adjusted their breathing by themselves, sat quietly in the chair, closed their eyes, and kept silent, while the surrounding environment was kept as quiet as possible. The duration of a single test was five minutes [42].

### Data analysis

We used the G*power software to calculate the statistical testing power of the required sample size, arriving at a sample size of 136 and an efficacy power of 0.99 based on an effect size of 0.25, an α of 0.05, three groups, two measurements. We otherwise used SPSS, version 21.0 (IBM, New York, USA) to conduct all statistical analyses.

For the normality testing, the Shapiro-Wilk test showed that all data were normally distributed (*P* > 0.05). Consequently, we used one-way analysis of variance (ANOVA) to analyse the difference in the demographic characteristics and repeated measurement ANOVA of 2 × 3 (time: baseline and week eight × group: PEG, ISMG, and CG) to analyse differences in sleep scores, BMI, and HRV between the groups. Finally, we used Pearson correlation coefficient to investigate the correlation between sleep, BMI, and HRV. We modified all our statistical results through the Greenhouse Geisser method and set the significance level at three levels, where *P* < 0.05 indicated low significance, *P* < 0.01 medium significance, and *P* < 0.001 high significance.

## RESULTS

### Changes in characteristics in sleep quality of children before and after intervention

**Figure 2:**
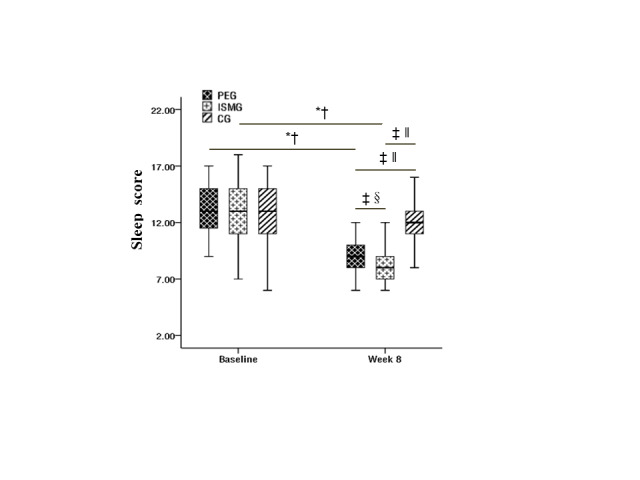
Box chart of changes in children's sleep quality before and after intervention. *Difference from baseline. †*P* < 0.001. ‡Difference between groups at week eight. §*P* < 0.01. ‖*P* < 0.001.

Repeated measure ANOVA for sleep quality showed significance for the the main effect of test time (F(1, 133) = 68.42; *P* < 0.001, η^2^ = 0.62); the main effect of groups (F(2, 133) = 18.44; *P* < 0.001, η^2^ = 0.22); and the interaction between time and groups (F(2, 133) = 36.61; *P* < 0.001, η^2^ = 0.36). Further simple effect analysis showed no significant difference among the three groups at baseline. However, the sleep quality in the PEG and ISMG were significantly lower than in the control group at week eight, as was the quality in the PEG compared to the ISMG. Meanwhile, the sleep quality scores in the PEG and ISMG were significantly lower than their baseline in the week eight, while there was no such difference for the control group (**Figure 2**).

### Changes in characteristics in BMI of children before and after intervention

**Figure 3:**
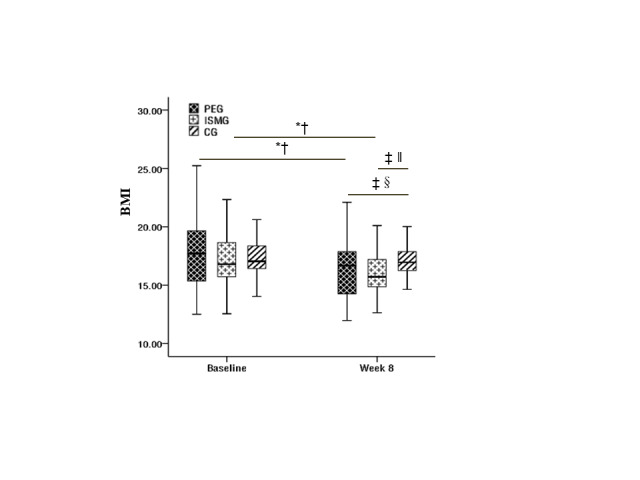
Box chart of changes in children's BMI before and after intervention. *Difference from baseline. †*P* < 0.001. ‡Difference between groups at week eight. §*P* < 0.05. ‖*P* < 0.01.

Repeated measure ANOVA for BMI showed significance for the main effect of test time (F(1, 133) = 56.55; *P* < 0.001, η^2^ = 0.30) and the interaction between time and groups was significant (F(2, 133) = 8.32; *P* < 0.001, η^2^ = 0.11), but no significance for the main effect of groups (F(2, 133) = 1.24; *P* > 0.05, η^2^ = 0.02). Further simple effect analysis showed that there was no significant difference among the three groups at baseline, but at week eight, the BMIs in the PEG and ISMG were significantly lower than the control group. Meanwhile, the BMIs in the PEG and ISMG were significantly lower than their baseline at week eight, while there was no difference for the control group (**Figure 3**).

### Changing characteristics of HVR in children before and after intervention

**Figure 4:**
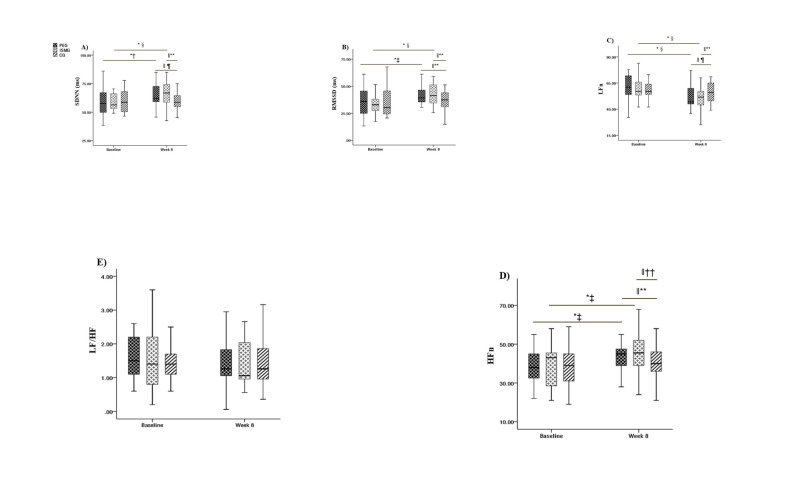
**Panel A.** Box chart of changes in children's SDNN. **Panel B.** RMSSD. **Panel C.** LFn. **Panel D.** HFn. **Panel E.** LF/HF before and after the intervention. *Difference from baseline. †*P* < 0.05. ‡*P* < 0.01, §*P* < 0.001. ‖Difference between groups at week eight. ¶*P* < 0.05, **P < 0.01. ††*P* < 0.001.

Repeated measure ANOVA for SDNN found significance for the main effect of test time (F(1, 133) = 15.28; *P* < 0.001, η^2^ = 0.10) and the interaction between time and groups (F(2, 133) = 3.87; *P* < 0.05, η^2^ = 0.05), but not for the main effect of groups (F(2, 133) = 1.61; *P* > 0.05, η^2^ = 0.02); (**Figure 4**, Panel A). Further simple effect analysis showed no significant difference among the three groups at baseline; however, the SDNN values were higher in the PEG and ISMG than the control group at week eight. Meanwhile, the values for PEG and ISMG were significantly higher at week eight than at baseline, while we observed no difference for the control group.

Repeated measurement ANOVA for root mean square value of the difference between RMSSD found that the main effect of test time (F(1, 133) = 18.64; *P* < 0.001, η^2^ = 0.12) and the interaction between time and groups (F(2, 133) = 4.05; *P* < 0.05, η^2^ = 0.06) were significant, while the main effect of the groups was not (F(2, 133) = 1.15; *P* > 0.05, η^2^ = 0.02) (**Figure 4**, Panel B). Further simple effect analysis showed no significant difference in RMSSD among the three groups at baseline; however, the values at week eight in the PEG and the ISMG were significantly higher than the control group, without significant difference between the PEG and the ISMG. Meanwhile, the PEG and ISMG were significantly higher at week eight than at baseline, while the control group showed no significant difference.

Repeated measure ANOVA for LFn showed significance for the main effect of test time (F(1, 133) = 26.77; *P* < 0.001, η^2^ = 0.17) and the interaction between time and groups was significant (F(2, 133) = 4.34; *P* < 0.05, η^2^ = 0.06), but not for the main effect of groups (F(2, 133) = 2.56; *P* > 0.05, η^2^ = 0.04); (**Figure 4**, Panel C). Further simple effect analysis showed no significant difference among the three groups at baseline, but detected significantly higher scores at week eight in the PEG and ISMG compared to the CG. Meanwhile, the PEG and ISMG had significantly higher values at week eight compared to baseline, respectively, while the control group had no significant difference.

Repeated measurement ANOVA for HFn found that the main effect of test time (F(1, 133) = 12.82; *P* < 0.001, η^2^ = 0.09) and the main effect of groups (F(2, 133) = 3.81; *P* < 0.05, η^2^ = 0.05) were significant, while the interaction between time and groups was not (F(2, 133) = 2.96; *P* > 0.05, η^2^ = 0.04) (**Figure 4**, Panel D). The post-hoc comparison showed no significant difference among the three groups at baseline; however HFn was higher in PEG and ISMG than the control group. Meanwhile, the HFn of the PEG and ISMG was significantly higher at week eight than baseline, respectively, while the control group showed no significant difference.

Repeated measurement ANOVA for LF/HF found that the main effect of test time and group, as well as the interaction effect of the test time and group, were not significant (**Figure 4**, Panel E).

### Relationship among sleep, BMI, and HRV of children after intervention

The Pearson correlation analysis showed that the sleep scores of children in the PEG were significantly negatively correlated with RMSSD and HFnorm and significantly positively correlated with LFnorm. Meanwhile, BMI was significantly positively correlated with sleep score and LFnorm, but significantly negatively correlated with HFnorm. In the ISMG, sleep scores of children after the eight-week intervention were significantly negatively correlated with RMSSD and HFnorm and were significantly positively correlated with LFnorm. Moreover, BMI was significantly positively correlated with sleep score and LFnorm, but significantly negatively correlated with HFnorm. In the control group, sleep scores of children after the eight-week intervention were significantly negatively correlated with RMSSD and HFnorm, but positively correlated with LFnorm. Lastly, BMI was positively correlated with sleep score and LFnorm (**Table 3**).

**Table 3:** Correlation between the main variables after intervention

Group	Variable	Sleep score	SDNN	RMSSD	LFn	HFn	LF/HF
PEG	Sleep score		0.10	−0.17*	0.19*	−0.20*	0.08
	BMI	0.22†	0.11	−0.09	0.17*	−0.18*	0.10
ISMG	Sleep score		0.09	−0.18*	0.20*	−0.21*	0.11
	BMI	0.25†	0.09	−0.12	0.19*	−0.17*	0.10
CG	Sleep score		0.05	−0.15*	0.16*	−0.14*	0.09
	BMI	0.16*	0.07	−0.08	0.15*	−0.12	0.04

BMI – body mass index, CG – control group, HF – high frequency, HFn – high-frequency norm power, ISMG, LFn – low-frequency norm power, RMSSD – root mean square value of the difference between adjacent R-R inter-periods, SDNN – standard deviation of continuous normal R-R inter-periods

*P < 0.05.

†*P* < 0.01.

## DISCUSSION

### Effects of an intervention integrating sports and medicine on children’s sleep

After an eight-week intervention, sleep scores of children in the PEG and the ISMG were significantly lower than those in the control group and were significantly lower at the last week than at baseline, indicating that both moderate-intensity physical exercise and an intervention integrating sports and medicine can effectively improve children’s sleep quality. This is consistent with previous studies. For example, Passos et al. [43] found that physical exercise, as one of the important non-pharmaceutical therapies, can be easily implemented and is an effective complementary alternative therapy for the treatment of sleep disorders. Another study has shown that, when the level of physical exercise was higher, adolescents had fewer night awakenings and insomnia symptoms, longer deep sleep, and better sleep quality [21], which was likely related to the exercise improving their psychology and increasing the secretion of melatonin [44]. This has been effectively verified in children, where physical exercise was found to increase the endogenous melatonin level of autistic children, thereby improving sleep quality [45]. Meanwhile, moderate-intensity aerobic exercise was found to be more effective in improving sleep disorders [46], possibly due to moderate-intensity exercise helping more effectively regulate the level of melatonin and the balance of the sleep-wake system, which is conducive to the improvement of sleep structure. Moreover, we also found that the sleep scores of children in the ISMG were significantly lower than those in the PEG, suggesting that the former intervention had a better effect on the improvement of children’s sleep quality. Research has reported that comprehensive health education was conducive to improving the sleep quality of patients with coronary heart disease [47], while health education combined with long-term aerobic exercise effectively improved the sleep quality of male depressed detoxics [48]. Therefore, we can hypothesise that the organic integration of medical elements (e.g. health knowledge education lectures) with physical exercise may be more effective in improving children’s subjective initiative for physical exercise and the awareness of the sleep shortage crisis, to improve their exercise efficiency and sleep quality. However, the circadian rhythm and the sleep structure of children differ between countries and regions, so the intervention implementation or testing should be controlled or adjusted accordingly to reduce the bias between different studies.

### Influence of an intervention integrating sports and medicine on children's BMI

We found that the BMI level of both the PEG and the ISMG significantly declined by week eight, indicating that the intervention was effective. Regular participation in physical exercise or activity an important way to control BMI and prevent obesity. Previous studies have shown that physical exercise could reduce the body fat composition of children [49]; that both the increase in physical exercise and the decrease in sedentary behaviour could effectively prevent the occurrence of obesity in children and adolescents [23], and that the decline of physical exercise level was the main cause of childhood obesity [50]. This suggests that physical exercise could regulate individual metabolic function, promote fat decomposition, and improve body composition [51]. Moreover, moderate to high-intensity physical exercise can effectively reduce the incidence of obesity [52] and the visceral fat content in the abdomen of obese women [53]. Based on physiological mechanism, moderate to high-intensity physical activity can generate more fast muscle fibres, cause muscle hypertrophy, increase muscle mass, and promote energy consumption during and after exercise [54]. This indicates that exercise intensity is a moderating variable for the improvement of BMI by physical exercise. Notably, we did not find a significant difference in BMI between the two intervention groups after the intervention, indicating that the adjustment effect of the intervention integrating sports and medicine on children’s BMI was not significantly better than that of a single physical exercise intervention. This may be because children are younger and that they do not pay much attention to their body management and appearance, coupled with their lacking understanding of health knowledge education and insufficient awareness of the harm of obesity, which reduces the effect of the intervention. This seems to be common to children worldwide, so it may also be true when tested in other cultural or geographical contexts.

### Influence of intervention integrating sports and medicine on children’s HRV

We found that the SDNN, RMSSD, and HFn were significantly increased in children in the PEG and ISMG at week eight, while LFn was significantly decreased. This is consistent with previous studies, such as tat by Guo et al. [55], who found that long-term adherence to Tai Chi could improve the overall regulatory function of the autonomic nervous system in elderly individuals, which manifested as a significant increase in the regulation effect of the vagus nerve and a decrease in the regulation effect of the sympathetic nerve. In testing a 12-week moderate-intensity cycling intervention in young men, Melanson et al. [56] found that HF, percentage of NN 50 in the total number of RR intervals (PNN50), and RMSSD were significantly increased after exercise, while the cardiac vagus nerve activity was enhanced. Meanwhile, studies haves shown that physical exercise can fully restore vagal nerve tone in obese children [17] and that different forms of physical exercise intervention could effectively improve the HRV of obese children and adolescents, thus improving the autonomic cardiac regulatory function [26]. In general, regular physical exercise could enhance the sympathetic and vagus nerve activities of individuals and promote the improvement of cardiac electrical stability, thus helping prevent malignant arrhythmias and other diseases [57]. However, we did not observe a significant difference in HRV between the PEG and the ISMG after the intervention, indicating that the intervention integrating sports and medicine is not significantly better than the single physical exercise in improving children’s HRV. This may be because routine medical interventions such as health education, medical monitoring, and evaluation cannot directly significantly impact HRV, so the dose-effect relationship needs to be further explored. Moreover, we found that the difference in LF/HF between the two intervention groups and the control group was not significant after the intervention, indicating that eight weeks of physical exercise and the intervention integrating sports and medicine can improve the autonomic nerve activity of children to a certain extent, and increase the leading role of parasympathetic nerve in sympathetic/parasympathetic balance. However, the changes in sympathetic/parasympathetic balance were not significant, which may be due to factors such as the shorter intervention period and the more active HRV level in children.

### Correlation among sleep, BMI, and HRV of children after an intervention integrating sports and medicine

We observed that, sleep scores, BMI, and HRV of children after the intervention in the three groups were correlated with varying degrees, and that the correlation coefficients in the two exercise groups were higher than those in the control group. In studies on sleep and BMI, sleep time of children and adolescents has been confirmed to have a linear relationship with their BMI to varying degrees [58,59] – that is, the lower the sleep efficiency or sleep quality, the higher the BMI, while sleep efficiency under 85% was a risk factor for obesity [21]. Wu et al. [60] also found that short sleep duration in children and adolescents was associated with obesity and other cardiovascular risk factors. This may be interpreted as a consequence of reduced sleep duration, increased levels of leptin and growth hormone-releasing peptide in blood, and leptin resistance in obese children with sleep deprivation, with lower sleep duration resulting correlating with more obvious leptin resistance [61]. Meanwhile, sleep duration was negatively correlated with the ratio of serum leptin to adiponectin in previous study [62]. In the study on sleep and HRV, Qi et al. [63] found that the sleep quality of athletes was positively correlated with HF and negatively correlated with LF/HF. When a person moves from wakefulness to sleep, parasympathetic tone increases, while breathing rate slows and becomes more regular. However, sleep deprivation could activate the hypothalamic-pituitary-adrenocortical axis, cause activation of the brain stem reticulum system, lead to hypothalamic excitation, and increase the daytime catecholamine level and glucocorticoid level, thus generating persistent sympathetic excitation, leading to increased heart rate, increased myocardial oxygen consumption, and coronary artery contraction [64]. Therefore, the sleep time of children and adolescents should be ensured to reduce the risk of cardiovascular diseases in adulthood [60]. In studies on BMI and HRV, childhood obesity was found to cause dysregulation of the autonomic nervous system (manifested by decreased HRV) and was associated with poor cardiovascular health [65,66], which was related to the decrease of the autonomic nervous activity or the increase of sympathetic nervous activity caused by obesity [67,68]. The results suggested that there was a close relationship between children’s health indicators, suggesting that an intervention integrating sports and medicine helps improve the sleep quality and BMI, and maintain the dynamic balance of HRV. However, due to differences in the mode of integration of sports and medicine in different countries or social backgrounds and the physiological characteristics of children, some results may be biased, so the application or test in other cultural or geographical contexts should keep the content of the intervention consistent and identify the physiological characteristics of participants.

### Limitations

In this study, we investigated and compared the effects of two intervention modes on children’s health. However, due to the relatively short intervention time, we did not find that the intervention integrating sports and medicine had more advantages in regulating children’s BMI and HRV. Further exploration should be made in the optimisation and design of interventions in the future, and long-term follow-up should be included to observe whether the effects are sustained. Meanwhile, since this study was preliminary and only examined the correlation between variables after the intervention, subsequent studies can reveal the causal correlation between variables by combining more physiological and biochemical indicators. Lastly, the period of rapid growth and development of our study participants (children aged 9–12 years) exposes our results to possible biases; we recommend appropriate control or adjustment in future similar studies.

## CONCLUSIONS

This study confirmed the positive impact of physical exercise on children's sleep, BMI, and HRV, and also clarified the improvement efficiency of an intervention integrating sports and medicine on children's health promotion. While the latter, integrated intervention has stronger potential to improve children's sleep quality, we observed no significant difference between the two intervention methods in BMI and HRV indicators. We speculate that the intervention integrating sports and medicine may be more conducive to improving individual psychological indicators rather than physiological indicators, which has a certain value for the formulation of follow-up intervention strategies for children's health promotion. We also found a significant correlation between sleep, BMI, and HRV, and observed that regular physical exercise or an intervention integrating sports and medicine can effectively strengthen this correlation. Researchers should further examine the synergistic benefits of different interventions on children's health promotion from a multi-dimensional rather than a single perspective.
